# Subregional Shape Alterations in the Amygdala in Patients with Panic Disorder

**DOI:** 10.1371/journal.pone.0157856

**Published:** 2016-06-23

**Authors:** Sujung Yoon, Jieun E. Kim, Geon Ha Kim, Hee Jin Kang, Bori R. Kim, Saerom Jeon, Jooyeon Jamie Im, Heejung Hyun, Sohyeon Moon, Soo Mee Lim, In Kyoon Lyoo

**Affiliations:** 1 Ewha Brain Institute, Ewha Womans University, Seoul, South Korea; 2 Department of Brain and Cognitive Sciences, Ewha Womans University, Seoul, South Korea; 3 Department of Neurology, Ewha Womans University Mokdong Hospital, Ewha Womans University School of Medicine, Seoul, South Korea; 4 Interdisciplinary Program in Neuroscience, College of Natural Sciences, Seoul National University, Seoul, South Korea; 5 College of Pharmacy, Graduate School of Pharmaceutical Sciences, Ewha Womans University, Seoul, South Korea; 6 Department of Radiology, Ewha Womans University College of Medicine, Seoul, South Korea; University of Medicine & Dentistry of NJ—New Jersey Medical School, UNITED STATES

## Abstract

**Background:**

The amygdala has been known to play a pivotal role in mediating fear-related responses including panic attacks. Given the functionally distinct role of the amygdalar subregions, morphometric measurements of the amygdala may point to the pathophysiological mechanisms underlying panic disorder. The current study aimed to determine the global and local morphometric alterations of the amygdala related to panic disorder.

**Methods:**

Volumetric and surface-based morphometric approach to high-resolution three-dimensional T1-weighted images was used to examine the structural variations of the amygdala, with respect to extent and location, in 23 patients with panic disorder and 31 matched healthy individuals.

**Results:**

There were no significant differences in bilateral amygdalar volumes between patients with panic disorder and healthy individuals despite a trend-level right amygdalar volume reduction related to panic disorder (right, *β* = -0.23, *p* = 0.09, Cohen's d = 0.51; left, *β* = -0.18, *p* = 0.19, Cohen's *d* = 0.45). Amygdalar subregions were localized into three groups including the superficial, centromedial, and laterobasal groups based on the cytoarchitectonically defined probability map. Surface-based morphometric analysis revealed shape alterations in the laterobasal and centromedial groups of the right amygdala in patients with panic disorder (false discovery rate corrected *p* < 0.05).

**Conclusions:**

The current findings suggest that subregion-specific shape alterations in the right amygdala may be involved in the development and maintenance of panic disorder, which may be attributed to the cause *or* effects of amygdalar hyperactivation.

## Introduction

Patients with panic disorder demonstrate a series of physiological and cognitive symptom clusters that are associated with recurrent and unexpected panic attacks, anticipatory anxiety, and phobic avoidance [1–4]. For instance, cardiorespiratory distress and dizziness-related symptoms were linked to the physiological domain of panic symptoms, while fear of dying and feelings of helplessness were more associated with the cognitive component [2]. Although the neurobiological underpinnings of panic disorder have not yet been established, the amygdala has received much attention as a critical brain structure involved in the pathophysiology of panic disorder [5–7]. Specifically, the amygdala may convey viscerosensory information from the thalamus to the locus coeruleus, periaqueductal gray region, hypothalamus, and parabrachial nucleus that may contribute to autonomic responses related to the physiological and behavioral arousal during panic attacks [6, 7]. Furthermore, the prefrontal cortex in combination with the hippocampus may play an important role in higher-level cognitive control over the amygdala, manifesting the cognitive component of panic symptoms [6, 7].

The model of fear conditioning and avoidance responses has been proposed to explain the pathophysiology of several anxiety disorders such as posttraumatic stress disorder, as well as panic disorder. Previous studies have suggested that the amygdala in connections with the prefrontal cortex, insula, and hippocampus plays an important organizing role in conditioned fear learning [8–10]. However, despite the analogy between panic disorder and fear conditioning and avoidance response model with regard to amygdalar involvement in core symptom manifestations, biological differences between these conditions should also be considered [6, 11]. For instance, panic attacks can spontaneously occur without prior exposure to any aversive stimuli [6, 11].

A series of clinical studies at rest as well as with provocation paradigms have been performed to examine the role of the amygdala-centered fear network consisting of the prefrontal cortex, hippocampus, hypothalamus, and brainstem in the development of physiological and cognitive symptom clusters of panic disorder. Earlier functional neuroimaging studies using positron emission tomography or single photon emission computed tomography at resting stage have demonstrated inconsistent findings regarding amygdalar activity in panic disorder [12, 13]. More recent resting functional magnetic resonance imaging studies have reported increased amygdalar activation during spontaneous panic attacks [14, 15]. However, findings on amygdala activation during provocation states with panicogenic challenges have been inconsistent. The amygdala activity during provocation was found to be greater [16], lower [17], or unchanged [18]. Decreased regional cerebral blood flow in amygdalar activity was suggested to be related to the cognitive component of panic symptoms such as anticipatory anxiety [17].

Amygdalar activation was rather consistently observed in response to emotional stimuli during fear conditioning [8], although some studies with methodological heterogeneity did not replicate this finding [8]. Interestingly, this amygdalar hyperactivation observed during fear conditioning may rapidly become habituated [19].

In contrast to seemingly inconsistent findings on functional imaging of altered amygdalar activity, structural imaging studies have demonstrated volume reductions in the temporo-limbic structures including the amygdala in patients with panic disorder as compared to healthy individuals [20–22]. Similarly, studies using the volumetric approach with manual segmentation have demonstrated amygdalar volume reductions in patients with panic disorder [23–25]. Although functional implications of smaller amygdala in panic disorder remain to be elucidated, smaller amygdalar volume is likely to be associated with higher anxiety levels [23].

Lesion studies have been performed to examine the role of the amygdala in fear conditioning. Patients with unilateral temporal lobectomy [26] or bilateral amygdalar damage [27, 28] showed impaired fear conditioning as compared with control subjects, implying the requirement of an intact amygdala for normal responses to aversive stimuli and then the acquisition of fear memory.

The amygdala is divided into a number of nuclei, which can be categorized to the laterobasal, centromedial, and superficial groups in human based on the cytroarchitectonically defined probability map of amygdalar nuclei [29, 30]. Amygdalar nuclei have differential connections with various cortical and subcortical structures and are known to have distinct roles in fear and anxiety [29, 30]. In this regard, subregion-specific deficits in the amygdala can be assumed to play a role in the development and maintenance of panic disorder [5]. It is important to note that amygdalar volume alterations related to panic disorder do not necessarily reflect equal deficits across the structure, but rather region-specific changes.

Notwithstanding the value of distinct function of amygdalar subregions, there have been surprisingly little research that has examined subregion-specific information on amygdalar alterations related to panic disorder. Only one study using voxel-based morphometry (VBM) has reported that smaller amygdalar regions that are associated with panic disorder may correspond to the central nucleus in the amygdala [23].

Recently, three-dimensional surface-based morphometric analysis of the amygdala has been employed in evaluating more subtle changes in shape composition, which would have been difficult to detect with traditional volumetric analysis [31–33]. Furthermore, local shape and size of the amygdala could be determined using the surface-based morphometric analysis, which can provide subregion-specific information [34]. However, to the best of our knowledge, structural alterations of the amygdala have not been evaluated in patients with panic disorder in terms of shape deformation.

In the current study, we examined the amygdalar volume differences between patients with panic disorder and carefully matched healthy individuals. In order to elucidate a specific role of the amygdalar subregions in panic disorder, three-dimensional surface-based morphometric analysis was also employed and used to determine the location and extent of the panic disorder-related amygdalar alterations. Furthermore, we examined whether potential subregion-specific alterations may be associated with panic symptom severity.

## Material and Methods

### Participants

Twenty-three patients with panic disorder and 31 age- and sex-matched healthy individuals participated in the study. Psychiatric diagnoses including panic disorder with or without agoraphobia were assessed using the Structured Clinical Interview for DSM-IV [1]. Severity of panic disorder was assessed using the Panic Disorder Severity Scale (PDSS)[35] and the Zung Self-Rating Anxiety Scale (Z-SAS)[36] only in patients with panic disorder to examine a relationship between the extent of amygdalar structural alteration and panic symptom severity. The Hamilton Depression Rating Scale (HDRS)[37] was administered to assess the presence of potential comorbid subclinical depression in both panic and control groups. Exclusion criteria were the presence of current or lifetime history of Axis I or Axis II psychiatric disorders other than panic disorder in the patient group, major medical or neurological disorders, pregnancy, or contraindication to magnetic resonance imaging including the presence of metal implants or claustrophobia.

All participants in the patient group have a history of taking psychotropic medications including antidepressants and anxiolytics. Participants in this study have been previously reported in other studies [38]. Demographic and clinical information regarding the study participants is presented in Table 1. This study was approved by the Institutional Review Board (IRB) at the Seoul National University Hospital and all participants provided written informed consent.

**Table 1:** Characteristics of Study Participants.

Characteristics			Patients with panic disorder (n = 23)	Healthy individuals (n = 31)	*p*
Age, mean (SD), y			32.0 (6.6)	30.9 (6.5)	0.56
Male, n (%)			13 (56.5)	18 (58.1)	0.91
PDSS score, mean (SD)			9.17 (6.15)	NA	NA
Z-SAS score, mean (SD)			42.8 (9.7) ^1^	NA	NA
HDRS score, mean (SD)			4.61 (5.09)	3.14 (4.32) ^2^	0.27
Psychotropic medications					
	*Antidepressants*, n (%)				
		Paroxetine	7 (30.4)		
		Fluoxetine ^3^	9 (39.1)		
		Mirtazapine ^3^	6 (26.1)		
		Sertraline	1 (4.3)		
		Bupropion	1 (4.3)		
	*Anxiolytics*, n (%)				
		Clonazepam			

SD, standard deviation; NA, not available; PDSS, Panic Disorder Severity Scale; Z-SAS, Zung Self-Rating Anxiety Scale; HDRS, Hamilton Depression Rating Scale

^1^ Data from one patient with panic disorder was not available.

^2^ Data from 3 healthy individuals were not available.

^3^ One patient was treated with both fluoxetine and mirtazapine.

### Image Acquisition and Amygdala Segmentation

All participants were scanned on a 3T GE whole body imaging system (GE VH/i, General Electric, Milwaukee, WI, USA). High-resolution three-dimensional T1-weighted images were acquired using a three-dimensional spoiled gradient echo pulse sequence with the following parameters: repetition time (TR) = 5.7 ms, echo time (TE) = 1.4 ms, inversion time (TI) = 400 ms, field of view (FOV) = 22 cm, matrix size = 256 x 256, flip angle (FA) = 20°. Axial T2-weighted images (TR = 3500 ms, TE = 118 ms, matrix size = 256 × 192, FOV = 22 cm, FA = 90°, slice thickness = 5 mm, 1.5 mm skip) and fluid-attenuated inversion recovery axial images (TR = 9900 ms, TE = 145 ms, TI = 2250 ms, matrix size = 256 × 192, FOV = 22 cm, FA = 90°, slice thickness = 5 mm, 1.5 mm skip) were also obtained and used to screen for possible gross structural abnormalities. A board-certified neuroradiologist who was blind to the subjects' condition examined all images.

**Fig 1:**
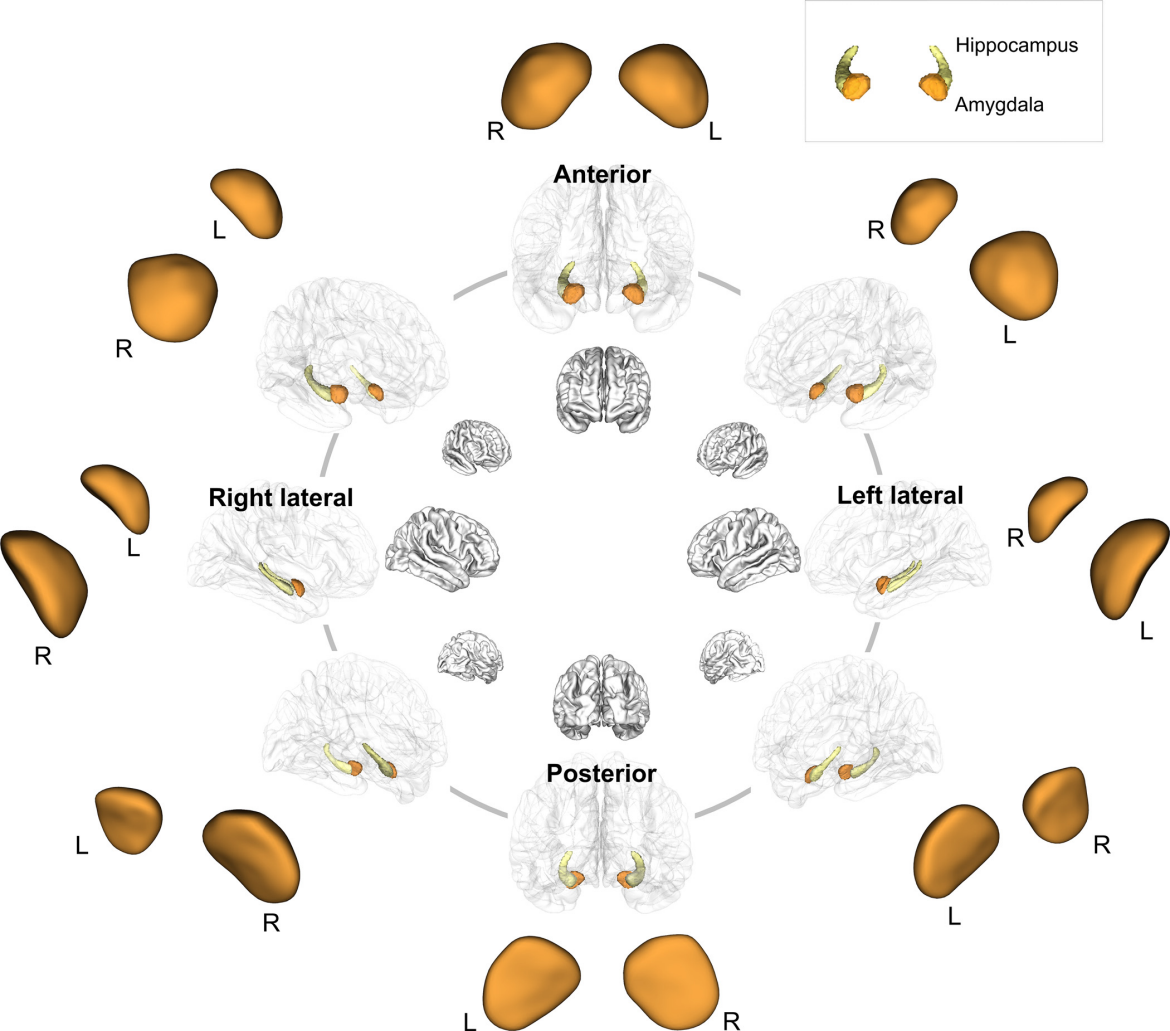
Anatomical location for the amgydala and hippocampus overlaid on the standard cortical surface and three-dimensional rendering of the amgydalar template. Abbreviations: R, right; L, left.

The amygdalae were segmented from the T1-weighted images using a rater-independent well-validated automatic segmentation method implemented in the FreeSurfer software (http://surfer.nmr.mgh.harvard.edu)[39, 40]. This segmentation method consists of several steps, in which T1-weighted images are first processed by performing motion correction and intensity inhomogeneity correction. After removing non-brain tissue, gray matter and white matter are segmented. Then, segmentation of subcortical structures including the amygdalae was performed using an atlas-based approach by assigning each voxel of the preprocessed volume to the corresponding probabilistic information on the amygdalae based on manually labelled training sets [41, 42]. An expert visually inspected the final segmented images to assure the appropriateness of the results. Manual editing was performed to correct mislabeled voxels or prominent anatomical errors if necessary. Anatomical location for the bilateral amygdalae are presented in Fig 1.

### Shape Analysis

The shape analysis of the amygdala was performed using the University of North Carolina spherical harmonic (SPHARM) shape analysis toolkit (http://www.nitrc.org/projects/spharm-pdm/)[43, 44]. In summary, the segmented bilateral amygdalar images were initially processed to fill any interior holes and minimally smoothed. These processed images were then converted into spherical coordinates, transformed into surface meshes, and then aligned. Spherical harmonic coordinates between corresponding vertices of surface meshes were computed by parameter-based rotation applying the first order ellipsoid from the spherical harmonic coefficients, which could eliminate the effects from the rotation and translation. Then, the spherical harmonic description was sampled into 1,002 triangulated surface points. Finally, the surfaces of the amygdala were spatially aligned to an averaged template using a rigid-body transformation [45]. A detailed description of the method is described elsewhere [32, 43, 44].

### Statistical Analysis

Demographic and clinical characteristics were compared between groups using independent t-tests or chi-square tests.

Multiple linear regression analysis was used to examine the effects of diagnosis on amygdalar volumes adjusting for age, sex, and intracranial volume. Effect sizes (Cohen's *d*) for group differences in bilateral amygdalar volume and shape were calculated.

**Fig 2:**
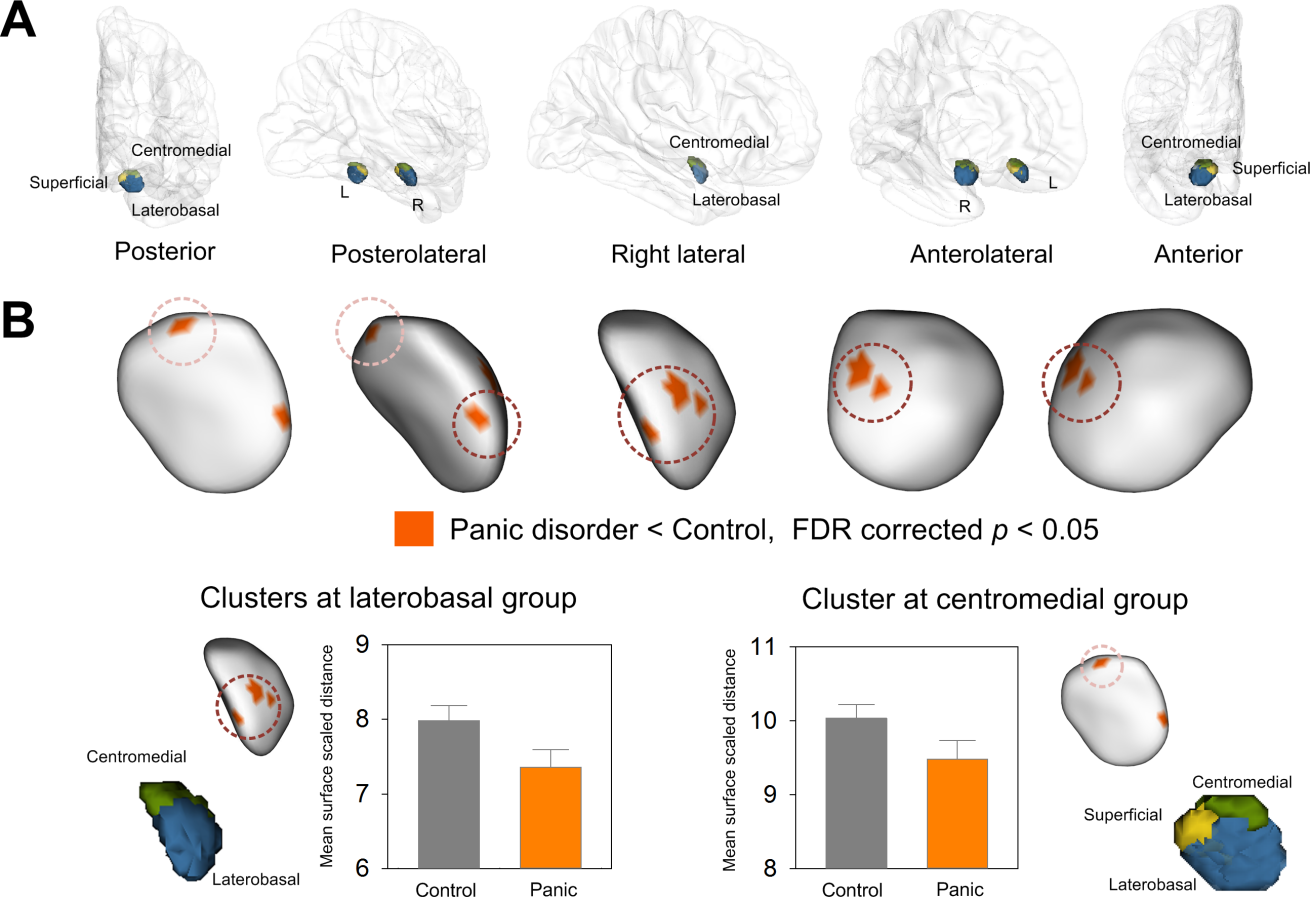
Clusters showing significant surface inward deformation in patients with panic disorder relative to healthy controls. (A) Surface rendering of cytoarchitectonically defined probabilistic maps of the superficial, centromedial, and laterobasal groups of the amygdala transposed onto the amygdalar template is presented. (B) Four clusters of inward deformation related to panic disorder at FDR corrected *p* < 0.05 are overlaid on the amygdalar template. Bar graphs show that mean surface scaled distance of clusters in the laterobasal and centromedial groups of the amygdala was lower in patients with panic disorder than in healthy individuals. Error bars indicate 95% confidence intervals. Abbreviations: FDR, false discovery rate.

Since the amygdala consists of structurally and functionally distinct subregions [5], local shape of the amygdala was investigated to determine the topological preference for potential diagnostic group effects on volumes. Euclidian surface distance was computed at every surface point and used as a dependent measurement for surface contour analysis. Multiple linear regression analysis was applied to estimate the diagnostic group effects on local surface distance at each triangulated point in the amygdala. Localization of the significant group effects on specific amygdalar subregions was performed using cytoarchitectonically defined probability map of amygdalar nuclei group [29]. In this cytoarchitectonically defined probability map, the amygdalar nuclei were divided into 3 groups including the superficial, centromedial, and laterobasal groups [29]. The superficial group included the anterior amygdaloid area, amygdalopyriform transition area, amygdaloid-hippocampal area, and ventral and posterior cortical nuclei. The centromedial group included the central nucleus and medial nucleus [29]. The laterobasal group consists of the lateral nucleus, basolateral, basomedial, and paralaminar nuclei. Surface rendering of amygdalar nuclei groups with 40% probability was performed using Slicer (http://www.slicer.org/) and each nuclei group was manually transposed onto the amygdalar template (Fig 2B)[46]. Individual significant vertices were localized to a specific nuclei group of the amygdala based on the highest probability of belonging to the laterobasal, superficial, or centromeidal groups. False discovery rate (FDR) was used to correct for the multiple comparisons.

To investigate the relationship between panic symptom severity and magnitude of surface alteration, mean Euclidian distance of individual surface points that belonged to the clusters showing significant diagnostic effects were calculated. Pearson correlation analysis was used to examine whether the severity of panic symptoms or anxiety symptoms was associated with the mean scaled distance of surface coordinates in each cluster in patients with panic disorder. All statistical analyses were performed using Stata SE, v11.0 (Stata Corp., College Station, TX).

## Results

Demographic and clinical characteristics are presented in Table 1. Patients with panic disorder and healthy individuals were well matched for age and sex. Among 23 patients, 7 (30.4%) patients had comorbid agoraphobia. All patients with panic disorder were treated with a combination of antidepressants and/or anxiolytics prior to the study enrollment. Detailed information on the medication history is shown in Table 1.

### Volume Analysis of the Amygdala

There were no differences in left amygdalar volume between patients with panic disorder and healthy individuals (*β* = -0.18, *p* = 0.19, Cohen's *d* = 0.45). However, although the difference did not reach statistical significance, patients with panic disorder showed a trend toward smaller right amygdala in comparison with healthy individuals after adjusting for age, sex, and intracranial volume (*β* = -0.23, *p* = 0.09, Cohen's *d* = 0.51). There were no differences in bilateral amygdalar volumes between panic disorder patients with agoraphobia and those without agoraphobia after adjusting for age, sex, and intracranial volume (right, *β* = 0.17, *p* = 0.44; left, *β* = -0.01, *p* = 0.95).

Bilateral amygdalar volumes were not associated with panic symptom severity measured using the PDSS (right, *r* = 0.06, *p* = 0.79; left, *r* = -0.01, *p* = 0.95) in patients with panic disorder. There was also no relationship between bilateral amygdalar volumes and scores of the Z-SAS (right, *r* = -0.11, *p* = 0.62; left, *r* = -0.08, *p* = 0.73) in patients with panic disorder.

### Shape Analysis of the Amygdala

**Fig 3:**
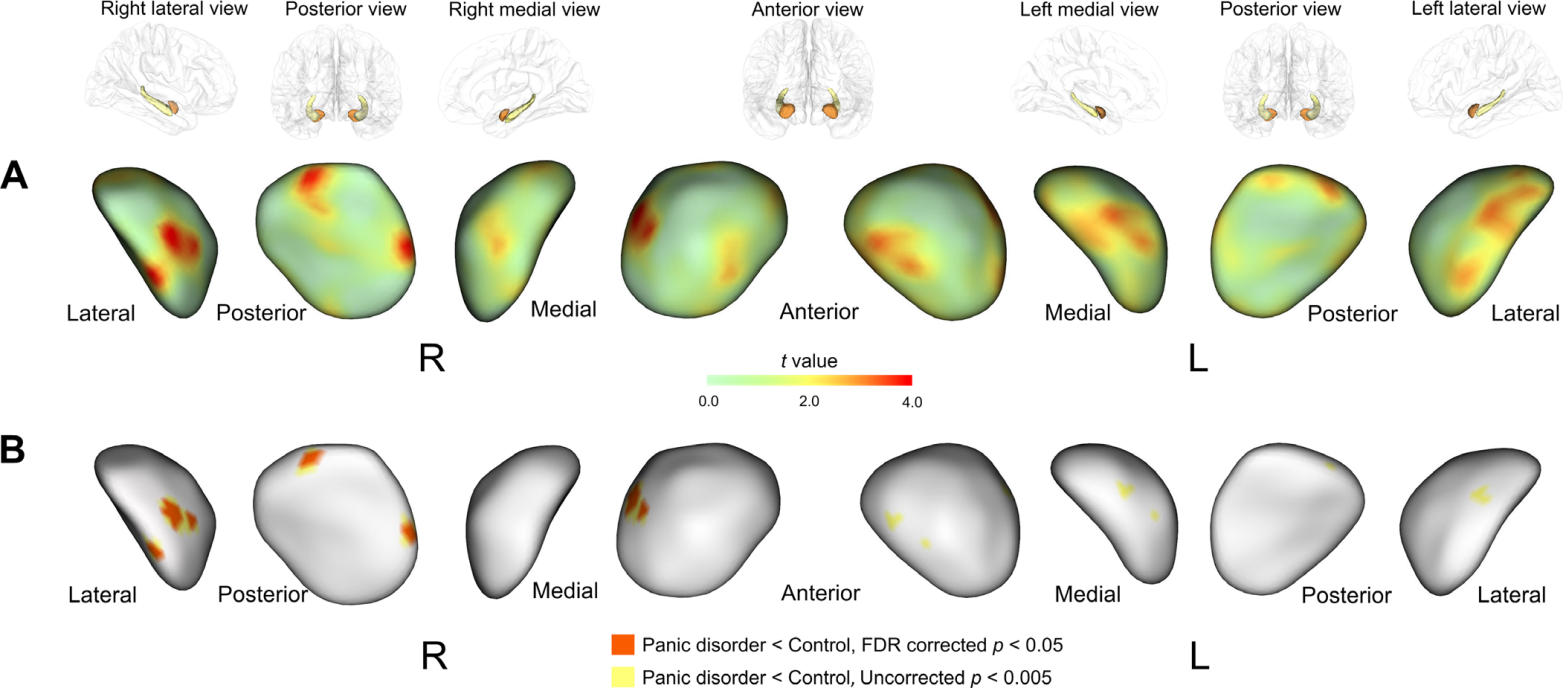
Statistical maps showing the location of shape difference in the amygdala between patients with panic disorder and healthy individuals. The *t* statistics (A) and probability (B) maps show the results of multiple linear regression analysis for estimating group effects on surface scaled distances of individual triangulated surface points after adjusting for age and sex. Abbreviations: FDR, false discovery rate; R, right; L, left.

Statistical maps for the diagnostic group effects on bilateral amygdalar shape are presented in Fig 3.

Inward deformation of the right amygdala shape was found in patients with panic disorder relative to healthy individuals. After correcting for multiple comparisons, the diagnostic group effects were significant in 4 clusters within the right amygdala, which were located in the laterobasal and centromedial groups (Fig 2). The effect sizes for inward deformation of the clusters located in the laterobasal and centromedial groups were 0.99 and 0.91, respectively (Fig 2B). Localized shape alterations were found in the left amygdala in patients with panic disorder compared to healthy individuals, although the difference was not statically significant after the correction for multiple comparisons (Fig 3). There were no differences in the mean scaled distances of clusters in the laterobasal (*β* = 0.10, *p* = 0.67) and centromedial (*r* = 0.24, *p* = 0.32) groups of the amygdala between panic disorder patients with agoraphobia and those without agoraphobia after adjusting for age and sex.

There were no significant relationships between panic symptom severity measured using the PDSS and mean scaled distance of clusters at both laterobasal (*r* = 0.16, *p* = 0.46) and centromedial (*r* = 0.33, *p* = 0.12) groups of the amygdala, respectively, in patients with panic disorder. In addition, anxiety symptom severity measured using the Z-SAS was not associated with mean scaled distance of clusters in patients with panic disorder (laterobasal group of the amygdala, *r* = -0.20, *p* = 0.38; centromedial of the amygdala, *r* = 0.40, *p* = 0.06).

## Discussion

Using three-dimensional surface-based morphometric analysis, we found surface deformation of the right amygdala related to panic disorder, specifically in the laterobasal and centromedial groups of the amygdala. In human, the laterobasal group of the amygdala consisting of the lateral nucleus, basolateral, basomedial, and paralaminar nuclei is anatomically located in the anterior or ventral segment of the amygdala, while the centromedial group of the amygdala including the central nucleus and medial nucleus corresponds to the posterior or dorsal segment [47]. This is the first in vivo evidence, to our knowledge, demonstrating that panic disorder-associated structural alterations in the amygdala may be localized and can occur in a subregion-specific manner. Shape alterations of the laterobasal and centromedial groups of the right amygdala were not associated with panic symptom severity.

Amygdalar volume reduction is one of the most replicated findings in patients with panic disorder in previous neuroimaging studies [5, 21, 24, 48]. In line with these findings, we found a trend-level volume reduction in the right amygdala in patients with panic disorder as compared with healthy individuals. Through its connections with a variety of other cortical and subcortical structures [49], the amygdala is involved in associative fear learning, processing emotional memory, and regulating autonomic responses to emotion, which are central to the pathophysiology of panic disorder [6]. Amygdalar subregions composed of a number of nuclei may have projections to different brain structures and have known to play distinct roles in emotional regulation [50]. While the role of specific amygdalar subregions have been extensively studied in animal models, there is emerging evidence from human studies suggesting subregional specificity of the amygdalar involvement in several anxiety disorders [51, 52].

In the current study, we found that panic disorder-related amygdalar structural alterations were prominent in the centromedial group including the central and medical nuclei, which can orchestrate fear responses and convey the viscerosensory information from the laterobasal group of the amygdala to the hypothalamus and brainstem [50, 53]. Consistent with this finding, one previous study that performed VBM analysis with a small volume correction using bilateral amygdalar masks has revealed panic disorder-related volume deficits in the right amygdalar subregion including central and medial nuclei [23]. Moreover, during panic attack, input information is recognized as fear, triggering the amygdalar nuclei of the centromedial group to evoke autonomic arousal and hypothalamic-pituitary-adrenal axis activation [6]. The amygdalar nuclei of the centromedial group, as the 'controller of the brainstem' [54], appears to be responsible for expression of fear and defensive behaviors [2, 55]. In the animal model, stimulation of the nuclei of the centromedial group may lead to a series of symptoms very similar to those during panic attacks [56]. Thus, structural abnormalities in the centromedial group of the amygdala may underlie increased and maladaptive behavioral responses to emotional stimuli in patients with panic disorder. Likewise, increased amygdalar hyperactivation in the safe condition relative to threat condition was reported in patients with panic disorder [57].

Another notable finding was that the areas of reduced amygdalar size in panic disorder patients relative to healthy individuals were primarily located in the laterobasal group of the right amygdala. The laterobasal group of the amygdala, as the 'sensory amygdala', includes lateral, basal, and paralaminar nuclei [30] and is responsible for emotional memory formation, fear conditioning, and representation of values [58]. Moreover, the primary function of the laterobasal group of the amygdala includes integration and higher level of neurocognitive processing of viscerosensory information through reciprocal connections with the cortical and subcortical regions, which were likely to be aberrant in panic disorder [5, 6]. Inferred from the animal model, the nuclei from the laterobasal group of the amygdala are expected to be involved in the pathophysiology of panic disorder. Likewise, repeated activation of the laterobasal group of the amygdala by blocking GABAergic inhibition in the animal model has been reported to lead to a constellation of symptoms frequently observed in panic responses [59]. However, this is the first human study, to our knowledge, to report structural deficits in the laterobasal group of the amygdala in patients with panic disorder.

It remains unresolved whether structural deficits in specific subregions including the laterobasal and centromedial group of the amygdala may be due to the result of repetitive activation during panic attacks or may cause repeated hyperactivation and resultant panic attacks [5]. It is important to note that subregion-specific shape alterations in panic disorder were likely to be independent of panic symptom severity. Similar to our findings, previous volumetric studies did not find relationships between amygdalar volume reductions and clinical measures including symptom severity [20, 24, 25]. Further longitudinal studies with a lager sample would be necessary to resolve this cause *or* effect issue of amygdalar deficits related to panic disorder.

Case studies of bilateral amygdalar damage which is caused by Urbach–Wiethe disease may provide important clues to the amygdalar role in panic disorder. Previous studies have demonstrated that a patient with bilateral amygdalar damage could not acquire conditioned responses to aversive stimuli [28] or recognize fearful stimuli [27]. These findings may suggest the essential role of the amygdala in the formation of fear memory. For instance, the presence of an intact amygdala may be required for normal fear conditioning. However, patients with bilateral amygdalar damage may experience not only spontaneous panic attacks [60] but also evoked ones by challenges with CO_2_ inhalation [61], implying that the structural loss of the amygdala may contribute to the development of panic attacks [61]. This could be further corroborated by our findings in combination with previous volumetric studies on the amygdalar structural deficits in panic disorder. Furthermore, considering the higher occurrence rate of panic attacks led by panicogenic challenges in patients with bilateral amygdalar damage, it can be assumed that an intact amygdala may contribute to the inhibition of panic attack [61].

In the current study, right amygdalar involvement was found in patients with panic disorder. This is concordant with previous volumetric studies reporting a greater effect size of right rather than left amygdalar deficits in panic disorder [20, 23–25]. Hemispheric dominance for fear processing of the amygdala may explain this finding of lateralized shape alterations in the right amygdala. Right hemispheric dominance for mediating emotional behavior and particularly for processing negative emotion has been reported [62, 63]. In addition, right and left hemispheres have been known to be involved in different aspects of emotion. For instance, the right and left amygdala may be responsible for modulation of acquired and innate fear, respectively [64].

There are several limitations to be considered in interpreting the current findings. The three-dimensional surface-based morphometric analysis may detect more subtle changes in shape that could otherwise be overlooked in volumetric analysis across the amygdala, as shown in the results of our current study. Our primary aim was to detect subregion-specific alterations related to panic disorder, but it should be noted the current sample size is relatively small to have sufficient power for detecting moderate effect size of volume difference in bilateral amygdalae (right, Cohen's *d* = 0.51; left, Cohen's *d* = 0.45) between patients with panic disorder and healthy individuals.

Since it is not possible to distinguish and segment amygdalar nuclei with structural MRI, allocation of shape alterations to specific subregions was performed provisionally based on cytoarchitectonically defined probability map of amygdalar subregions. Such indirect localization could not account for inter-individual variability of subregional morphometry.

Cellular and molecular mechanisms underlying amygdalar shape alterations could not be determined in this study. Furthermore, the current results could not account for a causal relationship between amygdalar atrophy and panic disorder. Our findings, however, imply that patients with panic disorder may exhibit subregion-specific shape alterations in the amygdala, which is, to the best of our knowledge, the first report in the human brain *in vivo*.

Although healthy individuals did not have any kinds of anxiety disorders based on the Structured Clinical Interview for DSM-IV, it should be noted that the PDSS and Z-SAS were not administered in healthy individuals to assess the presence of potential subclinical panic and anxiety symptoms.

In sum, three-dimensional surface-based morphometric analysis was first implemented in the present study to identify the specific location of focal changes in the amygdala related to panic disorder. Panic disorder-related structural alterations were found in the vicinity of the laterobasal and centromedial groups of the right amygdala, both of which together are responsible for the formation and modulation of emotional memory and fear responses. The current findings suggest subregion-specific involvements of the right amygdala in panic disorder. Future studies should address the issue of whether amygdalar subregional deficits reflect constitutional vulnerability to panic disorder or tear and wear caused by repeated activations during panic attacks.
